# Exploration of Hospital Inpatients' Use of the Verbal Rating Scale of Pain

**DOI:** 10.3389/fpain.2021.723520

**Published:** 2021-08-18

**Authors:** Luke Bosdet, Katie Herron, Amanda C. de C. Williams

**Affiliations:** ^1^Research Department of Clinical, Educational and Health Psychology, University College London, London, United Kingdom; ^2^Pain Medicine Department, Liverpool University Hospitals NHS Foundation Trust, Liverpool, United Kingdom

**Keywords:** pain measurement, pain assessment, pain communication, scale interpretation, analgesics

## Abstract

**Background:** Assessment of pain largely relies on self-report. Hospitals routinely use pain scales, such as the Verbal Rating Scale (VRS), to record patients' pain, but such scales are unidimensional, concatenating pain intensity and other dimensions of pain with significant loss of clinical information. This study explored how inpatients understand and use the VRS in a hospital setting.

**Methods:** Forty five participants were interviewed, with data analysed by thematic analysis, and completed a task concerned with the VRS and communication of other dimensions of pain.

**Results:** Participants anchored their pain experience in the physical properties of pain, its tolerability, and its impact on functioning. Their relationship to analgesic medication, personal coping styles, and experiences of staff all influenced how they used the VRS to communicate their pain.

**Conclusion:** Participants grounded and explained their pain in semantically similar but idiosyncratic ways. The VRS was used to combine pain intensity with multiple other elements of pain and often as a way to request analgesic medication. Pain scores need to be explored and elaborated by patient and staff, content of which will imply access to non-pharmacological resources to manage pain.

## Introduction

Both the original (1) and updated (2) definitions of pain make clear that the relationship between identifiable physical damage or pathology and the magnitude of pain is variable; pain cannot be directly observed or reliably estimated by clinicians. As a result, the preferred method of assessing pain in verbally competent patients is to use patient self-report. Thus, pain is “whatever the experiencing person says it is, existing whenever the experiencing person says it does” (3).

There are multiple methods used to assess pain; the most common are the numerical rating scale (4), the verbal rating scale (4), the visual analogue scale (5), and the McGill Pain Questionnaire (6, 7). None of the pain rating scales give instructions to indicate what pain phenomena are to be included, nor how they should be translated into the scale metric (8). They have different performance characteristics (9), with the former two more reliable than the latter (10). As they are accessible to verbal enquiry and response, they are therefore better suited to hospital settings (11). Despite superior reliability and validity of the numerical rating scale (12, 13), even in older patients (14), use of the verbal rating scale (VRS) is common. The VRS requires patients to rate their pain using ordinally-arranged adjectives describing pain intensity (e.g., no pain, mild pain etc.). Scores are assigned to the adjectives (e.g., no pain = 0, mild pain = 1 etc.), and are often treated as an interval or ratio scale (5) to enable quantitative description of pain and calculation of change with treatment.

However, treating verbal measures of pain in this way is controversial. Ordinal verbal categories provide no information about the distance between points on the scale (5) that would allow interval-level scoring, and the assumption that those distances are consistent across people in pain is untested. People vary considerably in how they convert their pain into verbal categories (15), and pain ratings are confounded by psychological and decisional processes that do not fit the linear structure necessary for equidistance (16). Further, single ratings do not separate the constructs of pain intensity, distress, and interference, when these are likely to be variably associated and idiosyncratically represented in a single term (17, 18). Of particular importance is the lack of separation of the sensory and affective components of pain (19, 20). Last, as noted by Fordyce (21), pain ratings are behaviours, so it is important to consider the context in which they are provided and the implications of the rating for both patient and receiver.

It can be advantageous to address pain intensity and pain distress separately in relation to clinical intervention (22), to avoid giving analgesic drugs for high pain ratings that in fact represent emotional distress (19). It is therefore helpful to understand how patients use the VRS to communicate their pain-related needs, and to use this to inform clinicians' responses. This study followed Uher's methodological guidelines (8) to explore how hospital inpatients translated their experience of pain into the VRS categories and how they communicated their pain needs to medical and nursing staff during routine pain assessments.

## Materials and Methods

### Procedure

Participants were recruited from adult inpatient wards in a central London hospital. NHS ethical approval was obtained (ID: 16/YH/0417) and as part of this process, an external “expert by experience” was consulted whose advice was used to make changes in the information sheets and protocols.

The researcher (LB) obtained an honorary contract with the hospital's specialist pain team who liaised with ward managers across the hospital for permission for ward staff to be approached about the study. Five wards agreed. The researcher then explained the study to the nurse-in-charge for that shift and obtained permission to collect data; the nurse-in-charge was asked to identify, and ask staff to approach, suitable patients based on the inclusion criteria: (a) over 16 years of age, (b) able to communicate effectively in English, and (c) with capacity to consent and take part. Eligible patients were then approached by the researcher. Data were collected across a period of 4 months in 2016–2017, with the process of asking permission and identifying patients repeated each day of data collection and on each ward.

Forty-five participants took part in the study. We intended to recruit patients equally across three groups: acute pain, chronic pain (longer than 3 months), and chronic with acute pain. However, participants' descriptions of their pain did not fit well into these groups so data on pain chronicity are provided. The study consisted of two parts: a semi-structured interview and a personal pain scale task. Both parts were conducted at the participant's bedside with their informed consent; we did not want to limit recruitment to patients who could walk to a private room, and few private spaces were available. Interviews were audio-recorded. Verbatim instructions are provided in Supplementary Material.

This hospital used a five-point VRS as the routine pain assessment for adults, with the categories of *No Pain, Mild, Moderate, Severe*, and *Very Severe* pain. The VRS was required by the hospital to be completed at the same time as other routine observations, usually every 4 h. Participant characteristics were recorded as they appeared in their medical notes: age, gender, ethnicity, and primary diagnosis (i.e., the reason for admission to hospital). For the sake of simplicity, comorbid diagnoses were not recorded. Participants were asked verbally about the length of time they had experienced pain.

### Interview Protocol

A semi-structured interview was developed to understand how inpatients used the VRS and the process by which they made their pain ratings. The interview started by asking participants to rate their current pain. The following questions broadly covered: (a) how participants understood the VRS categories, (b) how they selected a category, (c) how pain affected their emotions, (d) how they coped with their pain, (e) what they thought of the VRS, and (f) what else they would want to communicate to the hospital staff about their pain. Interviews consisted of nine core questions and the interviewer had the option to ask follow-up questions to elaborate or clarify on the above aims. Example questions from the interview protocol include: (a) For you, what are the main differences between mild and moderate pain? (b) What else would you like to tell the nurse or doctor about your pain? The full interview schedule and introduction can be found in the Supplementary Material.

The interview data were analysed using Thematic Analysis (23). The analysis was grounded in a critical realist epistemology in which the experience of pain was recognised as real and located in the body, but recognising that each individual constructed the experience in personal ways both in relation to him or herself and in communicating with others. This epistemological standpoint was chosen as it validated the participants' experiences as authentic, but recognised that communicating the experience is influenced by both individual differences and social processes. The iterative steps recommended by Braun et al. (23) were followed.

#### Transcription and Immersion of the Data

Each of the interviews was transcribed using Express Scribe Transcription Software. A total of 27 interviews were transcribed by the first author and the remaining 18 by a volunteer which were checked by the researcher against the audiotape for accuracy. The interviews were transcribed in accordance with recommendations in Barker et al. (24): verbatim speech content, but without information about the tone, loudness, speed etc. of speech. Aside from transcribing, all interview transcripts were re-read before beginning coding so the researcher would be familiar with the data.

#### Generating Initial Codes

The transcripts were uploaded into Nvivo, qualitative analysis software. The first author worked systematically through each of the transcripts, coding each unit of meaning found, and keeping as close to the original meaning as possible without implying any higher categorisation. All data were coded, without making assumptions of relevance to the research question to protect against the loss of potential themes or sub-themes at later stages.

#### Searching for Themes

The first author systematically worked through the codes of meaning to merge codes based on meta-level meanings from the explicit content of what the participant reported, rather than implicit or implied meaning. Previous theory also partly informed the type of codes that were chosen, in particular, that the pain experience can be divided into sensory, affective, and cognitive elements (4). For example, text coded as “stabbing,” “throbbing,” and “nagging” were coded under “Quality of Pain.” We also began to focus on the research aims and discarded some codes that were irrelevant to the study. For example, a participant who identified as an alcoholic was anxious that they would not be able to stop drinking.

#### Reviewing and Redefining Themes

We examined the developed themes against Patton's (25) criteria of internal homogeneity and external heterogeneity, in other words, whether the codes were sufficiently similar to constitute a wider theme, and whether the theme was different enough from other themes to be considered separately. For example, “Quality of Pain” was later absorbed into a broader theme of “Physical Properties of Pain.” This stage also involved credibility checks, described in the section below. Through this process the themes and subthemes evolved over several iterations before settling on the themes described in the **Results** section.

### Quality Evaluation

In keeping with guidelines for qualitative research by Elliot et al. (26), we included: (a) a “reflexive statement” reporting the researcher's theoretical and personal orientation; (b) a wide range of participants, described in terms of their pain and length of hospital stay, to improve the likelihood of developing a broad understanding of the phenomenon; (c) multiple participant quotations to illustrate each theme; and (d) credibility checks by analytical auditing and testimonial validity. Analytical auditing required another researcher to code five randomly selected transcripts, blind to the first coder's decisions, for comparison on development of initial themes. Testimonial validity, in the form of “synthesised member checking” (27), involved asking the original participants for feedback on the accuracy of the analysis.

#### Reflexive Statement

A reflexive position was taken in order to make more transparent the researcher's biases in analysis and interpretation. The first author and lead researcher is a male in his early thirties who was training in clinical psychology. He is from a working class family that generally considered post-modern epistemologies as irrelevant, in reaction to which he developed an interest in constructionism, but with a strong preference for pragmatism. His training in cognitive behavioural therapy and systemic approaches both emphasised splitting experience into different elements and sequences, while also recognising the often bidirectional nature of cause and effect. He was drawn to the topic of pain assessment mainly through dissatisfaction with what he perceived as oversimplification, as well as a desire to produce research with real world application.

### Personal Pain Scale Task

The purpose of this task was better understanding of how the VRS and elaborations of it described their experience of pain. Each participant was asked to elaborate their own personal pain scale using a horizontal line centred on a landscape A4 page as a template.

The general instructions to participants were to develop a scale that represented their pain. Participants were initially asked to record the VRS categories (*No Pain, Mild, Moderate, Severe* and *Very Severe*) on the line, spaced as made best sense to them, and then to add any terms they wished, located on the line. All terms were measured from *No Pain* (i.e., the left end of the line) and recorded in centimetres. Where participants did not indicate the exact position of a category on the line (e.g., they just wrote *Mild* above a section of the line), the position was calculated by the midpoint of the written word. During the task, participants were asked to “think out loud” and audio recorded in order to understand the method of development. The “thinking out loud” data were originally planned to be analysed in accordance with the method described under “Interview Protocol.” However, these data did not add any new substantial information in addition to the interview data, so were not included in this study.

We first examined whether participants placed the VRS categories in sufficiently similar positions to be considered a shared category, then examined the distances between categories, equidistance in particular, and finally we examined participants' additions and modifications to their scales.

## Results

### Participants

Forty-five participants (Table 1) completed the semi-structured interview and, of these, 29 agreed to complete the Personal Scale. Participants had a total of 25 different diagnoses, with the most common being Coxarthrosis (*n* = 9), Crohn's disease (*n* = 6), and fractures (*n* = 5).

**Table 1:** Participant sample characteristics.

**Characteristic**	**Frequency**
Gender	Male = 10; female = 35
Age	*M*: 50 (SD = 18); range: 19–81
Pain chronicity	*Mdn:* 6 years; range: 1 day−40 years
	*N* for pain < 1 year = 10
Ethnicity	White British: 28 (62%)
	White other: 5 (11%)
	Black or Black British: 4 (9%)
	Asian or British Asian: 1 (2%)
	Other: 1 (2%)
	Not Stated or Missing: 6 (13%)
Diagnostic category	Arthritis related disorders and problems: 17 (38%)
	Gastrointestinal problems: 17 (38%)
	Tumour related disorders: 3 (7%)
	Injuries and other disorders: 6 (13%)
	Missing: 2 (4%)
Recruitment wards	Orthopaedics: 21 (47%)
	Gastroenterology: 14 (31%)
	Oncology: 7 (16%)
	Short stay surgery: 3 (7%)

### Semi-Structured Interview

**Figure 1:**
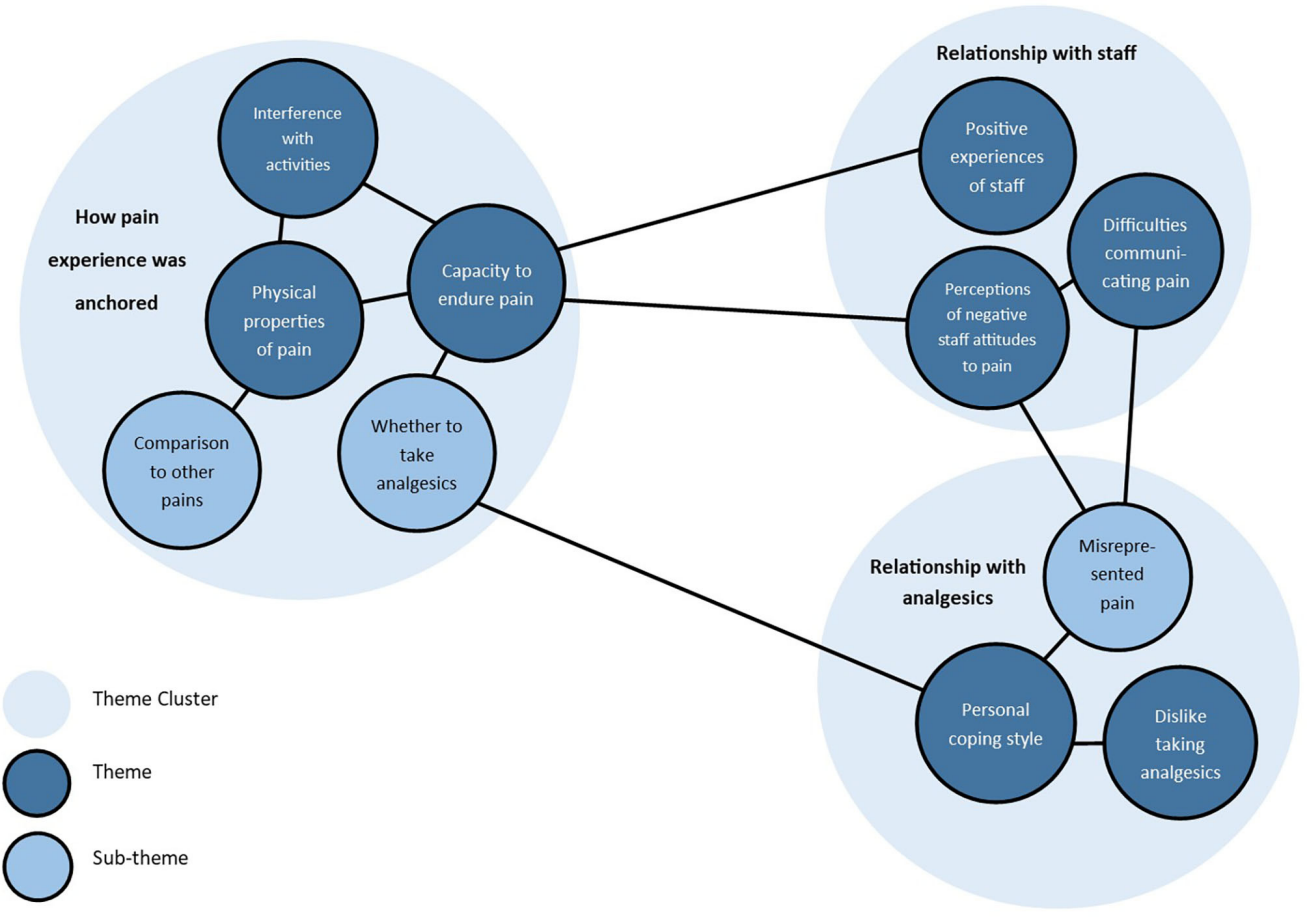
Theme map.

Analysis of the qualitative data from the semi-structured interviewed produced eight themes with three subthemes. These were grouped in three clusters: (a) how the pain experience was anchored, (b) relationship with analgesic drugs, and (c) relationship with staff. Figure 1 displays a map of the themes and relationships between them. The themes are explored below, highlighting similarities and differences between participants.

### Cluster 1: How Pain Experience Was Anchored

This cluster of themes pertains to how participants operationalised their pain in order to anchor the VRS categories, and included the physical properties of pain, how pain impacted on their function, their ability to endure pain, and how they coped with pain.

#### Theme: Physical Properties of Pain

Unsurprisingly, many participants (*n* = 25) made reference to the physical sensations of pain when demarcating categories of the pain measure. This included the amount of pain, number of pains, the longevity, constancy, and qualities of pain. Generally, as the number of these properties increased, reported pain severity worsened. However, the precedence and concatenation of these properties varied across participants. For example, pain longevity and constancy were sometimes given more prominence than the amount of pain.

*P14: I go back to the comparison with the broken leg and gastritis … Obviously, that hurt more than that … But this ultimately hurts more than that did because it's there all the time …*

Similarly, some participants commented on how the number of pains had an additive effect on pain ratings.

*P42: I don't just think of one pain I think of all my pain… and then amalgamate it according to how much, how much pain I'm in… if only one thing is hurting, then it will be a lower score than if my joints are very sore and I've got my pancreas kicking off, my bowels cramping…*

##### Subtheme: Comparison to Other Pains

Many participants compared current pain with other experiences of pain for their pain categories (*n* = 17), and with hypothetical pains. The time frame of these comparisons also varied, from the previous day in hospital to distant occasions. There were references to “everyday” or “normal” pains, as well as more exceptional pain from the past.

*P28: I'd compare [the current pain] to my kidney stones, I compare all my pain now to the worst pain I've ever experienced …*

*P12: … that would be stabbing pain I think. I mean I assume what you'd feel if you'd been shot …*

Comparisons to other pains also had emotional meaning.

*P45: today I'm feeling pretty good … but I feel a lot better because I was previously in quite severe pain*.

#### Theme: Interference With Activities

The majority of participants (*n* = 34), and the most prevalent theme in this cluster, described pain severity by referencing how much pain interfered with important activities. This includes mental activities, such as concentration and maintaining attention, as well as conversing with others, sleep, movement, and coping strategies. Participants described both what they could and could not do to delineate the severity of their pain.

*P44: I know [the pain is] there but I can also forget about it and focus on something else … I know I'm hurting but I know, I can do something else, you know, read, listen to something, the pain is not getting in the way of something else that I'm doing, that would be mild for me*.

*P2: ‘Mild', I can have a conversation with someone and completely focus on that conversation. ‘Moderate', my mind will start focusing slightly on the pain and I will lose the conversation slightly, or miss parts of what that person is saying, my concentration won't be as good. ‘Severe', I wouldn't be able to have a conversation*.

Participants reported using a wide range of coping strategies, the most common being focusing away from pain (*n* = 10), interacting with other people (*n* = 6), and physical activities such as going for walks (*n* = 6). With greater pain, participants reported being unable to use these strategies due to insufficient physical and mental resources, and hoped by reporting higher levels of pain to be given analgesics to help cope with it.

*P42: Normally I'm very good at distraction, mindfulness, that sort of thing … and if I can't use them, all I want is my medication*.

#### Theme: Capacity to Endure Pain

In addition to the physical qualities of pain and how it interfered with activities, participants also spoke about the tolerability of pain (*n* = 15). As pain became less bearable, severity of pain ratings increased.

*P1: Mild is something you can actually deal with …*

*P28: [Moderate pain is] probably stuck in bed but [I] can tolerate it …*

The *Very Severe* category was often described more elaborately compared to the other categories of the VRS. The words used often represented the limits of capacity, such as unbearable (*n* = 3), agony (*n* = 4), and excruciating (*n* = 1). Some participants reserved the *Very Severe* category for only the worst occasions and used it rarely (*n* = 7).

*P29: Oh, very severe is all-consuming, you can't think of anything, and when it gets like that yes I will, I do start crying and screaming … it is hell*.

*P10: whilst I'm in [very severe pain] I actually wish to die which is like, shocked me because normally I never do …*

Many participants commented on the emotional impact of being in pain. This included feeling low (*n* = 14), angry (*n* = 7), and anxious (*n* = 4) as a result of pain. The hospital environment also contributed to these emotions, with some participants stating that their usual coping mechanisms were constrained by the ward environment.

*P29: So yeah, [pain] controls everything with my emotions … When I'm having a bad time it turns me into a nasty, snappy, aggressive, horrible person and that's not who I am*.

Some participants described how emotions in turn affected how tolerable the pain was. Generally, negative moods exacerbated pain and reduced capacity to tolerate pain.

*P38: if you're getting a bit anxious and down with the pain then it's getting up to that severe level and you're having to ask for pain medication …*

##### Subtheme: Whether to Take Analgesics

One subtheme of this theme addressed whether participants would use analgesics (*n* = 27). In this sense, the VRS was used as a communication to nurses that the patient required analgesics. Some participants described a threshold at which they would begin to consider analgesics, mostly *Moderate* (*n* = 7) or *Severe* (*n* = 4). This consideration was related to the “Personal Coping Theme” in the “Relationship to Analgesics” cluster, in that the participant's approach to managing pain affected when they would use analgesics.

*P31: moderate pain is something that you kind of live with. Severe pain I guess you'd ring the call bell and say can I have [analgesic] please*.

This subtheme was also expressed as the effects of analgesics, in that pain became more tolerable.

*P38: I've always got a pain but [analgesics] will bring it down to a manageable level*.

### Cluster 2: Relationship to Analgesics

The second cluster concerns participants' mixed relationship with analgesics: welcoming help to cope with pain when other coping methods were not enough, but disliking the sense of dependence on analgesics or concerns about possible long-term effects of use. The need for analgesics strongly influenced how the VRS was used.

#### Theme: Dislike Taking Analgesics

Although all participants who reported pain said that they took some form of analgesic, many described aspects of analgesics that they disliked (*n* = 10), for reasons including side-effects, the build-up of tolerance, and fears of long-term damage.

*P2: I wonder, if everyone actually understood the severity of use, overusing painkillers and what it does to their body, if they would necessarily do that all the time*.

For those with chronic pain (*n* = 36), especially those with Crohn's Disease (*n* = 6) there was often conflict between adequate analgesia and sedation that impaired everyday life.

*P8: it may dissociate me from the pain but it doesn't help the pain itself … and I don't rate dissociation as help because I still want to be able to do what I want to do*.

*P2: it's got rid of my pain but I haven't gained anything from that, I've still lost my day*.

Another reason for disliking analgesics was the fear that analgesia prevented patients from checking their pain levels (*n* = 3), a concern they addressed by periodically stopping or refusing analgesics.

*P23: I need to know how bad the pain is, so if I'm junked up with painkillers I don't know*.

*P43: I am the type of person who from time-to-time will stop taking painkillers in order just to check [my pain]*

#### Theme: Personal Coping Style

Participants varied in their strategies for managing their pain and reporting pain levels to staff. Some participants had an uncomplicated approach to reporting their pain, preferring to give accurate responses when asked, and describing a straightforward relationship between their pain and the use of analgesics.

*P19: When it's there it's there, I always say it … I won't try to hide it*.

*P13: I'm in pain and I don't want to have a conversation about it, they're here and they know what to do and that's it*.

##### Subtheme: Misrepresented Pain

The VRS was widely understood as a way to communicate need for analgesia, but some participants described deliberately over- or under-reporting pain in order to influence the offer of analgesics. Many (*n* = 20) described under-reporting pain in the belief that they had a higher pain tolerance (*n* = 12), or in order to avoid making a negative impression on staff by appearing “soft,” a “nuisance” or a “wimp.” Two participants described how these attitudes developed from their families of origin.

*P11: I think potentially it could be cultural or generational as to why I don't think it's the done thing to say that I'm in pain … I grew up single parent family, mother who was extremely hard working and never complained a day … so it would for me feel wrong, I feel as though I'm moaning if I'm complaining …*

Some participants described a preference for handling pain using their own emotional coping methods, so under-reported pain in order to avoid discussions about analgesics.

*P8: I know that painkillers at that point aren't going to help, and my own techniques are going to be far superior so it's a lot easier to say I'm in no pain and get on with what I do*.

Deliberately over-reporting pain was much less frequently described (*n* = 4); participants described this as goal-orientated, most commonly to take control of when and what analgesics they received.

*P30: because by the time they actually go get the pain relief, they were only going to give me moderate pain relief like, it would have already turned into severe*

*P42: I can feel when my pain is progressing, and I like to pre-empt it before it gets to, before it gets too high. Because when it gets too high, it's then very very difficult to get back down again … So I might give a slightly higher pain score*.

### Cluster 3: Relationship With Staff

The themes in this cluster concern using the measure as a communication tool in an ongoing relationship with hospital staff. Participants discussed the difficulties of communicating their pain, as well as the positive effects of attentive staff.

#### Theme: Perceptions of Negative Staff Attitudes to Pain

Many participants described negative experiences with staff about their pain (*n* = 20), often suggesting disapproval of the use of analgesics or the report of pain. For example, participants related that some staff did not act on requests for analgesics, failed to pass on key information to other staff, or in one case directly refused to give prescribed analgesics. Several participants also described fears of being negatively evaluated by staff when asking for analgesics.

*P26: sometimes in the morning the doctors go ‘I gather you had a really good night' and you're like well, no, I told them I was in severe pain and that, so I don't think things get passed to the doctors unless they're really serious things*.

*Int: And do you think pain is taken seriously?*

*P26: Not really, no …*

Several participants described the problems caused by staff members' assumptions about what indicated pain (*n* = 5); this was a particularly prominent concern for participants with chronic pain problems, who noted that they do not always display their pain.

*P42: [The staff] criteria for severe is in tears, can't really communicate, asking for medication, and being kind of, having a face of, pulling a face … Making noises, that sort of thing, and if you're completely absent of that and you give an answer of severe then, I've had plenty of times where someone has said, but you look, you don't look like you're in severe pain, or they've kind of raised an eyebrow to sort of say, oh, oh yeah, course …*

*P8: You can't have pain if you're smiling, that would be a very good [laughs] assumption, if you're doing a crossword and listening to music you can't be in pain, when in fact that's exactly what I do when I am in pain*.

Many participants reported that staff used incorrect presentation of the VRS, using numbers instead of categories, or recording their own estimated pain levels without asking the participant.

*P42: quite often people will write down a score, but they haven't asked you. They haven't asked you what your pain is … I was finding that I was getting marks of, that said no pain, or moderate pain, or low pain … which isn't, isn't right*

*P8: my pain [has been] assessed in at least five different ways … I've been nought to four, one way, and nought to four the other way. Er, one to ten, ten to one, and the mild, moderate, severe but, again, on the ward I've never been asked until you said it if my pain was very severe. That's the first time I realised that was on the scale is when you said it …*

#### Theme: Difficulties Communicating Pain

Many participants remarked on the difficulties of communicating pain to staff, with or without the VRS (*n* = 27). On the VRS some participants struggled to distinguish between adjacent categories (*n* = 5). Participants also described the difficulty of converting the pain experience into scale categories.

*P14: I would just tell [staff asking on the VRS] I was completely unable to give an answer because I find the entire thing ridiculous … I don't think you can quantify pain when pain can mean so many different things …*

Two participants reflected on how difficult it was for staff to understand pain using medical knowledge and training; others commented more on the inadequacy of the scale in portraying pain. There was, nevertheless, some recognition of the subjective nature of pain and the difficulty for staff in understanding how people used the pain scale.

*P30: you think you know what pain is, like from what they teach in University, but it's nothing like that when you experience it yourself*.

*P28: so my pain to someone else's pain is going to be completely different, the way we rate it, so how is a nurse going to then be able to perceive that in terms on prescribing pain medication?*

#### Theme: Positive Experiences of Staff

The final theme of this cluster consists of how patients used the VRS in relation to positive experiences of relationships with staff (*n* = 10). Participants described how consistent and responsive care for their pain enabled them to report their pain needs more easily. For a few participants, this helped them overcome their usual stoic style which served as a barrier to requesting analgesics.

*P11: virtually everybody who I've come into contact with will ask me are you in pain? And they don't just ask are you in pain, they're asking using the scale, so you're getting used to the idea that it's not going to be a shock to say to somebody you're in pain*

*P15: people ask you, they ask very regular, that come and check on you, and they, they've very positive to you, you know, calling on the bell et cetera so you feel well cared … I wouldn't feel negative about saying well I am in pain*.

Another positive experience of staff was their demonstration that they observed non-verbal signs indicating pain.

*P29: But they know me well enough here that they can gauge my pain levels against what I'm doing …*

Two participants described how the attentiveness of staff made them feel more reassured and relaxed, which helped them deal with their pain.

*P17: I think that they know exactly what's going on with me, and, you know, where I should be and … I feel very sort of calm and relaxed about it …*

### Summary of Themes

How the VRS was used varied by participant across three main areas. Participants reified the categories in semantically similar but idiosyncratic ways. This included grounding the category demarcations using physical sensations, impact on functioning, and levels of tolerance. However, these demarcations also interacted with emotional state and current needs, such as sleep. The main use of the VRS reflected its use as communication, mainly expressing a need for analgesics. Individual participants' relationships to pain and analgesics played a key role in this communication, and positive and negative experiences of staff responses influenced this communication, enabling participants to communicate their pain needs or discouraging them from doing so.

### Personal Scale Task

Of the 45 participants interviewed, 29 (64%) agreed to complete the personal scale task, and 16 participants declined or were unable (e.g., due to poor eyesight, fatigue after the interview section). The 21 participants who recorded all five original VRS categories were included in the following analyses. The positions of all terms were normalised, such that 0 and 100 represented the two ends of the horizontal line. For example, *Severe* placed 18 cm from the left on a 26.8 cm line would be recorded as 67.2. This section first determined where categories were positioned by participants on the scale, whether the categories were positioned similarly by participants, and then tested the assumption that categories were equidistant.

**Figure 2:**
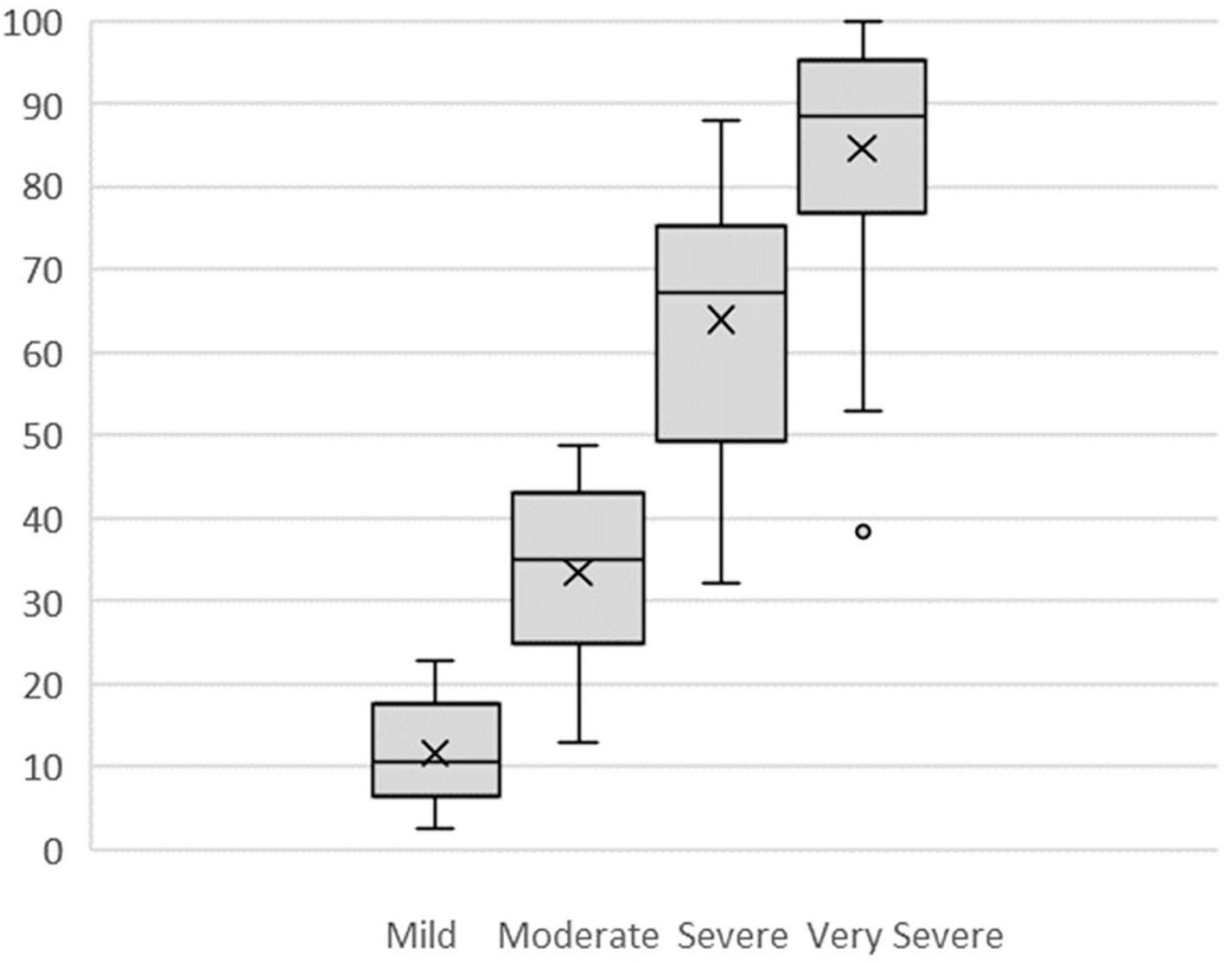
Box Plots of VRS mild, moderate, severe, and very severe numerical values assigned by participants. X is mean; ——— is median.

Figure 2 displays box plots for all four categories, *Mild* (*M* = 11.7, SD = 6.2), *Moderate* (*M* = 33.4, SD = 11.3), *Severe* (*M* = 63.9, SD = 14.6), and *Very Severe* (*M* = 84.6, SD = 15.6). All categories except *Very Severe* met assumptions for normal distribution. *Very Severe* was found to be significantly negatively skewed (z score = −3.34) and leptokurtic (z score = 3.16). Two scores in the *Very Severe* category were outliers (see Figure 2) with z scores > −2. Since the nature of this study was exploratory and did not assume normal distributions, these scores were retained and non-parametric tests used: a Kruskal–Wallis test and follow-up planned comparisons with Mann–Whitney tests. The four category positions were significantly different, *H*_(3)_ = 69.79, *p* < 0.001. *Mild* was significantly different from *Moderate, U* = 20, *z* = −5.04, *p* < 0.001; *Moderate* was significantly different from *Severe, U* = 22, *z* = −4.99, *p* < 0.001; and *Severe* was significantly different from *Very Severe, U* = 62, *z* = −3.98, *p* < 0.001.

To test the assumption of equidistance, the distance between each placed category on the scale was calculated for each participant who had recorded all five categories (*n* = 21). This created four distances: (1) *No Pain* to *Mild*, (2) *Mild* to *Moderate*, (3) *Moderate* to *Severe*, and (4) *Severe* to *Very Severe*. Distances were again normalised to 0 to 100 for comparison; for example, a distance *Mild* to *Moderate* of 6.6 cm on a 26.8 cm scale was recorded as 24.6.

*No Pain* to *Mild* was the smallest distance (*M* = 11.7, SD = 6.2), while *Moderate* to *Severe* was the largest (*M* = 30.5, SD = 10.1). *Mild* to *Moderate* (*M* = 21.7, SD = 9.6), and *Severe* to *Very Severe* (*M* = 20.7, SD = 8.6) were of similar size.

All four distances met assumptions for normality, so a one-way ANOVA was used to test the assumption of equidistance between adjacent categories. The overall result indicated significant differences: *F*_(3, 80)_ = 16.08, *p* < 0.001, and all but two *post-hoc* comparisons were statistically significant (see Table 2) using a Bonferroni adjusted alpha level of 0.008 (0.05/6). Overall, the assumption that there are equal distances between pain categories was not supported. In particular, there is a large difference between *Moderate* and *Severe*.

**Table 2:** Category distance comparisons.

**Comparison**	**Statistics (*t* test, *p*-value, effect size)**
No pain to mild and mild to Moderate (*M* = 11.73) (*M* = 21.71)	*t*(20) = −3.92, *p* = 0.001, *d* = 1.23
No pain to mild and moderate to severe (*M* = 11.73) (*M* = 30.48)	*t*(20) = −7.08, *p* < 0.001, *d* = 2.24
No pain to mild and severe to very severe (*M* = 11.73) (*M* = 20.65)	*t*(20) = −3.65, *p* = 0.002, *d* = 1.20
Mild to moderate and moderate to severe (*M* = 21.71) (*M* = 30.48)	*t*(20) = −2.81, *p* = 0.011
Mild to moderate and severe to very severe (*M* = 21.71) (*M* = 20.65)	*t*(20) = .37, *p* = 0.714
Moderate to severe and severe to very severe (*M* = 30.48) (*M* = 20.65)	*t*(20) = 3.18, *p* = 0.005, *d* = 1.05

### Additions to and Modifications of the Scale

**Figure 3:**
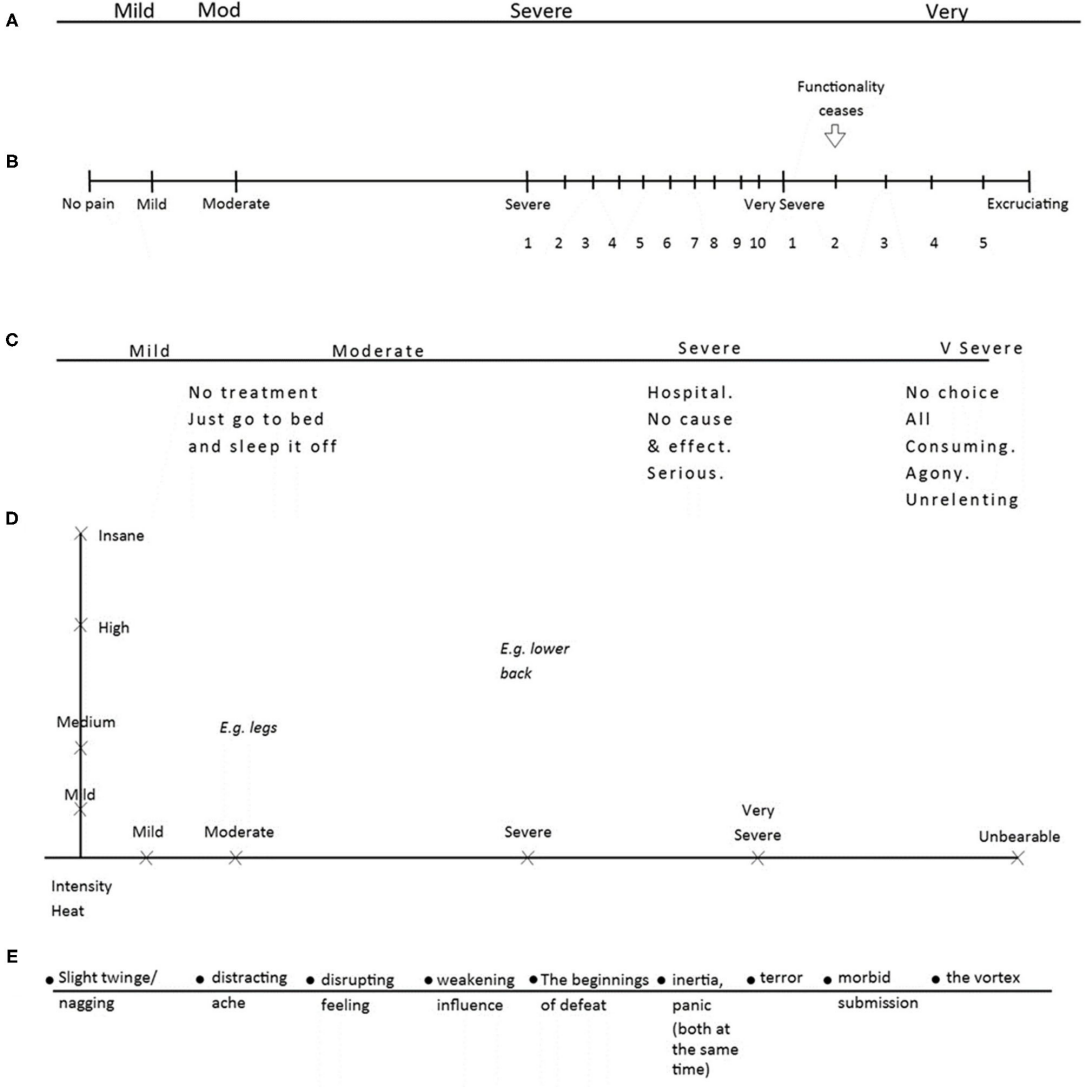
Five personal scales. Five personal scales: **(A)** P22's scale, **(B)** P42's scale, **(C)** P11's scale, **(D)** P20's scale, **(E)** P14's scale.

Of the 29 personal scales elaborated by participants, four had no changes or additions to the VRS. Four participants chose to expand the VRS categories but did not add any new ones. Sixteen participants added their own categories to the VRS, and two created a completely new set of categories. Three participants made major structural changes to the scale. Overall, every scale was unique in representing the participant's relationship with pain. Some representative examples of each type of change are displayed below (Figure 3).

Figure 3A shows P22's scale (*Very Severe* has been shortened to “*Very*”). This participant had a very short recent experience of pain and chose not to make any additions or changes to the scale. In contrast, P42 (Figure 3B) reported a longer experience of pain and had used of the scale over many years. This participant's personal scale was superimposed over the *Severe* and *Very Severe* categories in the form of a numerical scale, converted back to VRS terms when answering medical staff. P11 (Figure 3C) also chose to expand on the existing categories, but by adding interventions that might be required and personal experience or evaluation of that pain. P20 (Figure 3D) altered the scale completely by adding a y axis of “Intensity/Heat” to represent the partial independence of these aspects in their nerve pain; they also used the two-dimensional space to map different pain locations, as pain often varied across their body. Last, P14 (Figure 3E) replaced the VRS categories that did not describe their experience of pain with their own descriptions of feelings and experiences of pain.

## Discussion

This study explored how hospital inpatients understood and used a VRS pain scale, presented routinely for monitoring. Overall, participants described a rich variety of meanings in their communication of pain, and reporting pain was heavily influenced not only by social and emotional factors but also specifically by participants' perceived need for analgesics and likelihood of the staff providing them. A large proportion of the interviews were spent discussing analgesic medication, despite there only being one question in the interview protocol. The results from this study support assertions that patients combined pain affect with other pain elements in their ratings on unidimensional pain scales (19, 22), and made comparisons with previous pain experiences and reported pain in idiosyncratic ways (17) in a complex decision process (16). Two themes in particular are similar to those described by Robinson-Papp et al. (18) with outpatients: the multiple influences on pain rating, and the individuality of referents for the anchor points. The distances between categories, derived from representing the verbal descriptors in spatial terms, corroborate previous findings that categories are not equidistant (4), as they would be were the VRS an interval scale.

Consistent with other research about low adherence to pain management protocols (28, 29), this study also found that participants reported multiple instances of improper use of the pain scale by staff, such as completing it without consulting the patient. This may reflect poor training, weak adherence or inadequate implementation of assessment policies (30), or other organisational or practical issues that influence use of the scale by staff (31, 32). Since this study did not sample staff experience, explanations can only be speculative. Nonetheless, the findings reported here extend the known difficulties with pain management protocols by describing some of the impact these behaviours have on patients. These included a reluctance to report pain due to a fear of being adversely judged as a person, with overall detriment to clinician understanding of the patient. It was encouraging to obtain accounts of positive experiences, of feeling “cared for,” enabling participants to report their pain and, to some extent, to manage it themselves. Similarly, staff should be aware of the different ways that pain can be expressed, especially in chronic pain patients, and not believe that it can be determined simply by global impression of behaviour, mood or facial expression.

This research elaborated on the way that pain ratings from unidimensional pain scales such as the VRS, but also including numerical rating scales and visual analogue scales, combine multiple elements of the pain experience, including pain affect, disability, coping and magnitude, in an ordinal but non-linear and idiosyncratic fashion. To turn to analgesics for all of these, expressed in high pain ratings, is clearly ineffective. While there are more detailed pain measures, such as the McGill Pain Questionnaire (7) that attempt to segregate the various components of pain, they are not practical for routine hospital care. High ratings on a verbal or numerical scale should instead invite further questions to determine what intervention or support would be most helpful. Repeated and consistent use of the unidimensional scale with a follow-up exploration of support options would allow staff and patients to develop expertise in managing pain.

The finding of uneven distances between categories of the verbal rating scale means that interval-level scoring is inappropriate, and some categories, particularly “*Moderate*,” may represent a wide span of intensities, overlapping with adjacent categories. In clinical settings this may mean that some changes are more meaningful than others. For example, a pain rating increase of moderate to severe could represent a greater increase than mild to moderate. Although numerical and spatial scales avoid this problem, change on any unidimensional scale may represent increase or decrease in pain severity or improvement or deterioration in other functions such as mood, mobility, or sleep.

Participants incorporated their capacity to endure pain in the categories they chose, but that capacity was fluid, varying with context and emotional states. Addressing the emotional needs of patients is likely to be a more useful intervention than analgesics when emotional contexts make pain difficult to manage. In particular, feeling low and anxious were the most frequently reported emotional consequences of pain, and these may respond to support for coping, clarifying expectations of pain, providing information about pain, validating pain and providing reassurance. Likewise, consistent and responsive care by staff helped patients cope with the anxiety-provoking nature of pain and the hospital environment.

Similarly, some of the VRS use was goal-orientated. For example, people reported higher pain levels at night, when pain might interfere with sleep, in order to request analgesics. Staff should be aware that if pain is interfering with a valued activity, pain levels are likely to be rated higher. It may be useful to explore this with the patient, aiming for problem-solving. Equally, participants often described keeping occupied as a way to cope with pain, and providing the means to do so, such as liberal visiting hours, can help them to use this strategy.

A strength of this study was the examination of the VRS in an ecologically valid setting. It showed that when staff requested a pain rating, it was often perceived by patients to be a question about whether they required analgesia. This may be a feature of the ward environment and system; the measure is probably used rather differently in a research setting.

### Limitations

The study has several limitations. Interviews took place at the bedside in open wards, without confidentiality, and this may have discouraged participants from disclosing sensitive issues, such as distress or loneliness. Second, there are limits to the accuracy with which people can describe their decision-making processes, being unaware of unconscious biases and subject to self-presentation to the researcher as an honest witness. Third, potential participants were identified by the nurse-in-charge as suitable, in order not to disturb those who were too ill or cognitively impaired to consent or participate, but this may have skewed selection toward more articulate or amenable patients, or those more likely to give a good account of their interactions with staff. The participant group was mainly white British and female, and so may underrepresent male viewpoints or those associated with particular ethnic groups. This may be particularly relevant in the approach to coping with pain, where culture and gender roles influence social expectations and norms, and affect preferences. However, the study has strengths in representing a range of patient diagnoses, time in pain, and ages.

### Conclusion

Inpatients using the VRS combined multiple dimensions of pain in idiosyncratic ways, including sensory, affective, cognitive and functional dimensions. Each participant made sense of each VRS category, and the distances between categories, in unique ways. The VRS was widely used as a tool to express need for analgesics, and scores were adjusted according to the participant's wish for analgesia and expectations of staff. These results have implications for staff training in using the pain scale and interpreting scores, and in involving patients in this process. Pain scale ratings should not be assumed to represent simple pain intensity and need further investigation in setting such as this where they are widely used for monitoring care.

## Data Availability Statement

The raw data supporting the conclusions of this article will be made available by the authors, without undue reservation.

## Ethics Statement

The studies involving human participants were reviewed and approved by National Health Service IRAS project ID: 16/YH/0417. The patients/participants provided their written informed consent to participate in this study.

## Author Contributions

LB carried out the empirical work, wrote a long version for his doctoral thesis, and helped prepare this version for publication. AW was principal supervisor for the empirical work and wrote the first draft of the paper from LB's thesis. KH assisted the empirical work as external supervisor, and was involved in rewriting this version from the thesis.

## Conflict of Interest

The authors declare that the research was conducted in the absence of any commercial or financial relationships that could be construed as a potential conflict of interest.

## Publisher's Note

All claims expressed in this article are solely those of the authors and do not necessarily represent those of their affiliated organizations, or those of the publisher, the editors and the reviewers. Any product that may be evaluated in this article, or claim that may be made by its manufacturer, is not guaranteed or endorsed by the publisher.
