# Supplementary material for: Exploration of Hospital Inpatients' Use of the Verbal Rating Scale of Pain
Source: Front Pain Res (Lausanne). 2021 Aug 18;2:723520. doi: 10.3389/fpain.2021.723520 (PMC8915699; doi:10.3389/fpain.2021.723520)
Supplement: Supplementary file 1 [file Data_Sheet_1.DOCX]

**Interview Schedule**

**Introduction**

This section sets the scene for the interview and expectations for the participant. This is based on Smith, Flowers and Larkin (2009).

1. Introduce project, enquire about interest and present information and consent forms
2. Allow the participant to read forms alone (allow 10 minutes) and return to answer any questions
3. If the participant agrees to continue, remind participant of participation rights and discuss confidentiality depending on interview location
4. Set up expectations about interview itself by saying something similar to*:*

*Thank you agreeing to take part in this interview. As we have just discussed, we are interested in your own experience and opinions about assessing pain while on the ward. There are no right or wrong answers, and you can elaborate on your answers as much or as little as you like. The conversation may be one sided as I may say very little. Some of the questions may seem obvious as I am trying to get to grips with how you understand things. I might interrupt you at times to keep the interview on track or ask questions about topics that come up that seem important for this research.*

**Rating pain**

During recruitment it is planned that a member of the medical team (referred to as nurse throughout this document) will ask the patient about their pain. This helps make the pain rating as ecologically valid as possible. If for some reason this does not occur, the interviewer can ask this themselves.

- *If the nurse was to come over to you now and ask you to rate your pain on a verbal scale from no pain, minor pain, moderate pain, severe pain, or very severe pain, what answer would you give?*

X refers to the answer given.

Z refers to answers higher or lower on the rating scale that are used as comparisons during the questions.

**Interview Questions**

This section outlines the questions to be asked of the participant. It is interested in determining how they use the pain scale and communicate their needs to health care professionals.

- *How did you come to answer X to the nurse? On what basis did you answer X?*
- *For you, what are the main differences between X and Z? (*This question can be repeated for other points on the verbal scale if it is considered useful) *How would you know if you felt Z?*
- *Has there been a time when you felt similar pain to now but gave a different answer? Why?*
- *How does the pain affect how you feel emotionally? Does this affect what pain rating you give to the nurse?*
- *What do you think about the pain scale they use?*
- *What else would you like to tell the nurse or doctor about your pain? What else do you think they would need to know?*
- *In an ideal world, what are all the things that would help your pain?*
- *Do you think that an analgesic/pain killer would help your pain? In what way/how much?*

**Written Task Instructions**

This section introduces the hand written task. The task is verbally introduced as described in italics below.

*Pain is a complex experience, and scales like the one you were asked about earlier (rating pain as mild, moderate, severe or very severe) can seem too simple for what you want to tell medical and nursing staff about your pain. So this is a chance to show how you would like your pain to be assessed.*

*Please have a look at this piece of paper* (This will be a landscape A4 page with a line running through the centre). *As you can see, there is a line running through the middle of the page. You can add your own terms for pain, as well as placing the ones we already use (No pain, Mild Pain, Moderate Pain, Severe Pain, Very Severe Pain) on the scale where you think they belong. I would like you to talk out loud while you do this so I can understand your thinking process.*

*As an example, If we asked people to make a rating scale for how hot things feel, offering them the terms “very cold, cold, warm, hot” they might place them like this (*draw them on the example sheet along with brief ‘thinking out loud’ demonstration*). Then we might want to add our own terms* (draw freezing, chilling, boiling etc.)

*Did you have any questions?* (Once participants understand the task then proceed) *The rating scales are: no pain, mild pain, moderate pain, severe pain, and very severe pain. Remember to say out loud what you are thinking while you complete the task.*

(Once completed) *Are there any other terms you want to include on the page?*

*Thank you very much for your*
